# Editorial: Computational approaches to neurodegenerative diseases

**DOI:** 10.3389/fncom.2026.1952655

**Published:** 2026-08-26

**Authors:** Milos Antonijevic

**Affiliations:** Department of Informatics and Computing, Singidunum University, Belgrade, Serbia

**Keywords:** artificial intelligence (AI), computational modeling, digital biomarkers, machine learning, neurodegenerative diseases

Neurodegenerative and neurological diseases are characterized by complex interactions between molecular pathology, neural dysfunction, systemic risk factors and gradual shifts in cognition and behavior. Their diverse presentation, frequently inconspicuous in early illness, continues to challenge prompt diagnosis, prognosis and therapy planning. Computational techniques are becoming crucial in tackling this challenge by integrating diverse data, discovering relevant biomarkers, simulating disease dynamics, and enabling clinically interpretable decision-making.

The Research Topic *Computational approaches to neurodegenerative diseases* features five papers that showcase the scope of current computational research in this topic. The contributions span mechanistic mathematical modeling, medical image classification, electroencephalographic analysis, quantitative gait assessment, and prediction of diabetes-related neurological complications. Collectively, they demonstrate how computational methods can connect biological understanding with practical diagnostic and risk-stratification tools.

Two contributions focus on Alzheimer's disease classification using complementary neurophysiological and neuroimaging modalities. Anicin et al., in “*Metaheuristic-driven dual-layer model for classifying Alzheimer's disease stages*,” propose a two-tier framework in which convolutional neural networks extract informative representations from magnetic resonance images, followed by classification using XGBoost and LightGBM models. Metaheuristic optimization is used to refine the model configurations, while explainable artificial intelligence methods provide insight into the features influencing individual predictions. By addressing multiclass differentiation among stages of Alzheimer's disease, the study moves beyond binary disease detection and emphasizes the clinical importance of estimating disease progression.

Wang R. et al., in “*Feature fusion and WOA-GWO optimization for Alzheimer's disease detection with sparse EEG channels*,” examine the potential of electroencephalography as a non-invasive and comparatively accessible diagnostic modality. Their framework combines non-linear dynamic EEG features with a hybrid Whale Optimization Algorithm and Gray Wolf Optimizer for channel selection. The reported results indicate that strong classification performance can be retained with only a small subset of EEG channels. This reduction is particularly relevant for the development of lightweight, portable, and computationally efficient systems that may be more readily translated into clinical practice.

These studies explore data-driven recognition of Alzheimer's disease, but Mehmood et al. focus on the underlying dynamics of Alzheimer's disease in “*A Physics Informed Neural Network (PINN) framework for fractional order modeling of Alzheimer's disease*.” The authors create a fractional order mathematical model including neurons, amyloid beta, tau proteins and microglial responses. Fractional derivatives allow to describe memory and history-dependent processes that are difficult to express by standard integer-order models. Then, a physics-informed neural network is employed to approximate the system dynamics respecting biological and mathematical constraints. This work demonstrates how mechanistic knowledge and machine learning can be combined, particularly when observations are noisy or limited, and highlights the potential of computational models for evaluating therapeutic control strategies.

The Research Topic also extends beyond Alzheimer's disease to neurological conditions associated with vascular and metabolic dysfunction. Wang Y-Y et al., in “*Development and internal validation of a spatiotemporal gait parameter-based diagnostic model for cerebral small vessel disease*,” integrate quantitative gait parameters with conventional clinical characteristics. Their model identified sex, hypertension, body mass index, stride length, step frequency and step width as meaningful factors to discriminate between patients with cerebral small artery disease and healthy controls. The work demonstrates the possibility of quantifiable behavioral and motor features as digital biomarkers that might provide assistance for screening in settings where sophisticated neuroimaging is not accessible or feasible.

Tie et al., in “*Diabetes Duration and HDL Cholesterol as Dominant Predictors of Diabetic Neurological Complications: A Parsimonious Risk Model from the UK Biobank*,” investigate the prediction of diabetic neurological complications using clinical, physical activity, and proteomic data. Machine learning and survival-analysis methods are combined with SHAP-based interpretation to identify the most influential predictors. A central finding is that a simplified model based on diabetes duration and HDL cholesterol achieved performance comparable to a considerably more complex model. This result highlights an important principle in clinical computational modeling: increased model complexity does not necessarily produce greater clinical value. Parsimonious models may offer improved interpretability, generalization, and implementation, although external validation remains essential.

Several common themes emerge across these contributions. First, computational modeling is most informative when it is founded in variables that have clinical or biological value. Second, feature and channel selection may lower the measurement and computing burden, without necessarily reducing predictive performance. Third, explainability is increasingly becoming an important need rather than an optional add-on, especially for systems meant to assist high-stakes healthcare judgments. Finally, the studies underline the need of balancing prediction accuracy with practicality, resilience and interpretability.

Important challenges remain. Many computational models are developed using retrospective, single-center, or publicly available datasets and therefore require prospective and external validation. Differences in acquisition protocols, population characteristics, disease definitions, and preprocessing procedures may limit generalizability. Future research should aim at multicenter assessment, transparent reporting, calculation of uncertainty, model calibration, and direct comparison with established clinical procedures. Combining longitudinal neuroimaging, electrophysiological, genetic, motor and clinical data might further improve individualized models of illness development.

Taken together, the articles in this Research Topic demonstrate that computational approaches can contribute at multiple levels, from modeling pathological interactions to extracting biomarkers and constructing clinically applicable prediction systems. Continued collaboration among computational scientists, neuroscientists, clinicians, and data specialists will be essential for transforming these methodological advances into reliable tools for earlier detection, improved monitoring, and individualized management of neurodegenerative and neurological disease.

